# The Potential Effects of Sensor-Based Virtual Reality Telerehabilitation on Lower Limb Function in Patients with Chronic Stroke Facing the COVID-19 Pandemic: A Retrospective Case-Control Study

**DOI:** 10.3390/medsci13020065

**Published:** 2025-05-23

**Authors:** Mirjam Bonanno, Maria Grazia Maggio, Paolo De Pasquale, Laura Ciatto, Antonino Lombardo Facciale, Morena De Francesco, Giuseppe Andronaco, Rosaria De Luca, Angelo Quartarone, Rocco Salvatore Calabrò

**Affiliations:** 1 IRCCS Centro Neurolesi Bonino-Pulejo, 98124 Messina, Italy; mirjam.bonanno@irccsme.it (M.B.); paolo.depasquale@irccsme.it (P.D.P.); laura.ciatto@irccsme.it (L.C.); antonino.lombardo@irccsme.it (A.L.F.); giuseppe.andronaco@irccsme.it (G.A.); rosaria.deluca@irccsme.it (R.D.L.); angelo.quartarone@irccsme.it (A.Q.); roccos.calabro@irccsme.it (R.S.C.); 2 Department of Clinical and Experimental Medicine, University of Messina, Piazza Pugliatti, 1, 98120 Messina, Italy; morenadefrancesco00@gmail.com; 3 Department of Biomedical, Dental Sciences and Morphological and Functional Images, University of Messina, 98122 Messina, Italy

**Keywords:** sensor-based telerehabilitation, virtual reality, chronic stroke, lower limbs, COVID-19, neurorehabilitation

## Abstract

Background/Objectives: Individuals with chronic stroke often experience various impairments, including poor balance, reduced mobility, limited physical activity, and difficulty performing daily tasks. In the context of the COVID-19 pandemic, telerehabilitation (TR) can overcome the barriers of geographical and physical distancing, time, costs, and travel, as well as the anxiety about contracting COVID-19. In this retrospective case-control study, we aim to evaluate the motor and cognitive effects of balance TR training carried out with a sensor-based non-immersive virtual reality system compared to conventional rehabilitation in chronic stroke patients. Methods: Twenty chronic post-stroke patients underwent evaluation for inclusion in the analysis through an electronic recovery data system. The patients included in the study were divided into two groups with similar medical characteristics and duration of rehabilitation training. However, the groups differed in the type of rehabilitation approach used. The experimental group (EG) received TR with a sensor-based VR device, called VRRS—HomeKit (n. 10). In contrast, the control group (CG) underwent conventional home-based rehabilitation (n. 10). Results: At the end of the training, we observed significant improvements in the EG in the 10-m walking test (10MWT) (*p* = 0.01), Timed-Up-Go Left (TUG L) (*p* = 0.01), and Montreal Cognitive Assessment (MoCA) (*p* = 0.005). Conclusions: In our study, we highlighted the potential role of sensor-based virtual reality TR in chronic stroke patients for improving lower limb function, suggesting that this approach is feasible and not inferior to conventional home-based rehabilitation.

## 1. Introduction

Stroke is a leading cause of disability, significantly impacting the daily lives of survivors. Individuals with chronic stroke often experience various impairments, including poor balance, reduced mobility, limited physical activity, and difficulty performing daily tasks [1]. Although these challenges are more pronounced in the acute stage, they can persist for years. Factors, such as lower limb muscle weakness, abnormal muscle tone, sensory deficits, and impaired vision and spatial awareness, can contribute to mobility and balance deficits, increasing the risk of falls, which can lead to severe complications and consequently, hospitalization [1]. In particular, impaired motor control and weakness of lower limbs not only reduce the muscular force but also affect the inter-limb coordination [2]. Muscle strength of the affected lower extremity is usually reduced by 34–62% in patients post-stroke compared to healthy individuals [3]. As a result, enhancing physical activity and improving balance, muscle strength, and mobility in this population is crucial. To promote functional recovery and maintain progress, individuals with chronic stroke are required to continue constant rehabilitation or exercise interventions [4]. However, several factors can hinder adherence to rehabilitation after discharge from the hospital. For example, therapist availability, program costs, family support, and long distance to transportation could limit access to the continuity of care rehabilitation programs [5]. In this context, home-based telerehabilitation (TR) offers a remote solution to guarantee the continuum of care, enabling stroke survivors to maintain long-term exercise routines at home while also alleviating the workload on the therapist [6]. TR can enhance access to specialized rehabilitation services for individuals facing geographic or transportation challenges [7]. In the context of the COVID-19 pandemic, TR can overcome not just the barriers of geographical and physical distancing, time, costs, and travel, but also the anxiety about contracting COVID-19 [8]. Beyond the pandemic situation, TR offers the potential to bridge care gaps and deliver rehabilitation services anytime and anywhere. This is particularly beneficial for patients who live in rural or underserved areas, or those who face challenges accessing rehabilitation facilities due to mobility issues or multiple health conditions [9,10].

Conventional rehabilitation to improve balance and lower limb strength is focused on Bobath concept therapy, which includes exercises to train static and dynamic postural responses as well as core stability muscles and isometric muscle contraction exercises [4,11]. However, this approach is therapist-dependent and rates of adherence to such interventions are typically low because conventional exercises tend to be repetitive and unattractive [5]. On the other hand, innovative rehabilitation devices, like virtual reality (VR) and exergaming, can offer real-time augmented feedback while an individual performs specific motor tasks [12]. The audio-visual biofeedback can enhance motor learning while individuals perform specific motor tasks [13]. Audio–visual biofeedback not only improves motor learning but also stimulates cognitive engagement, such as attention, working memory, and executive functions [14]. These cognitive processes are crucial for motor recovery, as they help individuals focus on tasks, process feedback, and adapt their movements effectively [15]. Furthermore, VR environments can create complex, multisensory scenarios that challenge motor and cognitive functions, promoting neuroplasticity [12]. This holistic approach is particularly relevant to stroke patients, in whom motor and cognitive deficits often co-occur, making it essential to address both aspects during rehabilitation [16]. The integration of motor and cognitive rehabilitation through VR allows for a more complete recovery path, improving not only physical abilities but also enhancing cognitive resilience by fostering neuroplasticity and cognitive resilience [17,18].

In the context of TR devices, the Virtual Reality Rehabilitation System (VRRS) HomeKit device (Khymeia, Padua, Italy) enables the patient to carry out the training program at home, completely supervised by the therapist through a remote workstation [19]. The TR tool is equipped with VR that provides repetitive and task-oriented motor training, with exercises that gradually increase difficulty, promoting the motivation and overall functioning of the patient [19].

In this retrospective case-control study, the main purpose of our study is to investigate the potential effects of sensor-based virtual TR on lower limb functions in chronic post-stroke patients. As a secondary aim, we also seek to explore the impact of this training on global cognitive functions.

## 2. Materials and Methods

### 2.1. Study Population

Twenty chronic post-stroke patients, who attended the Robotic and Behavioral Neurorehabilitation Unit of the IRCCS Centro Neurolesi “Bonino-Pulejo” between May 2020 and June 2021, underwent evaluation for inclusion in the analysis through an electronic recovery data system. During this period, the use of TR increased significantly at our facility due to the impact of the COVID-19 pandemic.

This retrospective case-control study adhered to the principles of the 1964 Helsinki Declaration and received approval from our Research Institute Ethics Committee (ID: IRCCSME 39/2024-Tele-ictus).

The retrospective design of the study, along with data extraction from electronic medical records, helped reduce scoring bias. Motor and cognitive criteria were used to select appropriate post-stroke patients for inclusion in the analysis. Patients were included if they underwent TR using a sensor-based VR system or a traditional home-based rehabilitation approach. Retrospective evaluations, conducted at the onset and conclusion of training, were carried out by a multidisciplinary rehabilitation team comprising a neurologist, physiatrist, physiotherapist, and psychologist.

Inclusion criteria were as follows: (i) ischaemic or haemorrhagic chronic stroke (<1 year), (ii) haemodynamic stability, (iii) age range between 18 and 75 years, (iv) the presence of a stable internet connection, and (v) the presence of a caregiver able to use simple digital devices. Otherwise, patients’ exclusion criteria were (i) severe cognitive and behavioral impairments, (ii) cardiorespiratory instability or other medical illness potentially interfering with treatment, (iii) severe limb spasticity (Modified Ashworth Scale—MAS score > 3), and (iv) a high risk of spontaneous fracture.

### 2.2. Data Collection

Demographic and clinical information was retrospectively gathered from all patients. The outcomes, along with details of the rehabilitation sessions, were recorded and analyzed. Although the data were collected between May 2020 and June 2021, this study adopted a retrospective case-control design. Consequently, after the conclusion of the rehabilitation interventions, we retrospectively evaluated which patients met the strict inclusion and exclusion criteria and could be appropriately matched between the two groups. The data extraction, verification of eligibility, and matching process required a thorough clinical review, which contributed to the delay in finalizing the manuscript. Before participating, patients gave general informed consent for the use of their data in research.

### 2.3. Procedures

The patients included in the study were divided into two groups with similar medical characteristics and rehabilitation training duration. However, the groups differed in the type of rehabilitation approach used. The experimental group (EG) received TR with a sensor-based VR device, called VRRS—HomeKit (n. 10), while the control group (CG) underwent conventional home-based rehabilitation (n. 10). Our rehabilitation protocol consisted of 40 training sessions, each lasting around an hour for both groups (i.e., five sessions per week for eight weeks, by our established standard and clinical research protocols). All patients were evaluated before (T0) and after (T1) the treatment with clinical scales by a skilled physiotherapist not involved in the training and by a psychologist.

Moreover, both patients and caregivers of EG participated in three meetings with the physiotherapist, who followed the patients in TR, to receive essential guidance on properly using the tool. Following these sessions, patients engaged in three simulation training sessions over two weeks, with each session lasting approximately one hour. The simulations were conducted using the Tele-Cockpit workstation and the same VRRS HomeKit device that patients used later at home. If the caregiver or patient felt uncertain about operating the system, additional instruction sessions were scheduled to provide further assistance. During face-to-face meetings, the caregiver and the patient were provided with personal protective equipment (gloves, an FFP3 mask, a disposable gown, boots, and a cap) and their temperature was measured. Each caregiver was also provided with a list of appropriate behaviors in the hospital context, as indicated by the sanitary direction, including not removing the individual protection devices.

Both groups performed a rehabilitation program that, although primarily focused on lower limb recovery, also included exercises targeting trunk control and postural stability. These components were intentionally integrated to support balance and gait performance, which are functionally interrelated with lower limb function [20,21] in post-stroke patients (Table 1).

### 2.4. Outcome Measures

Each post-stroke patient was evaluated at T0 and at T1 through clinical scales to assess global lower limb functions and cognitive impairment. In particular, motor evaluations included the following: a 10-m walking test (10MWT) [22] to assess walking or gait speed in meters per second over a short distance, and it can be employed to determine functional mobility and gait; Timed-Up-Go (TUG) [23], which is used to determine fall risk and measure the progress of balance, sit to stand, and walking functions. On the other hand, cognitive outcomes measures included the Montreal Cognitive Assessment (MoCA), which is a cognitive screening test questionnaire used in the detection of mild/severe cognitive impairment (cut-off < 26), assessing attention, concentration, executive functions, memory, language, visuospatial skills, abstraction, calculation, and orientation.

### 2.5. Conventional Home-Based Rehabilitation

Conventional home-based rehabilitation consists of a motor face-to-face setting at patients’ homes. During the study period, patients were followed at home by a physiotherapist in a face-to-face modality. During training sessions, the physiotherapist and the patients, where both the patient and the physiotherapist were in close contact, wore FFP3 masks. However, only the physiotherapist wore disposable gowns, shoe covers, and sterile gloves to minimize the risk of COVID-19 infection. The rehabilitation treatment was focused on active-assisted and active exercise for lower limbs in addition to balance exercises (e.g., standing up or sitting down, changing direction, and speed). During all sessions, post-stroke patients were manually guided and supervised by the physiotherapists to prevent falls (see Table 1).

### 2.6. Sensor-Based Non-Immersive VR TR

**Figure 1:**
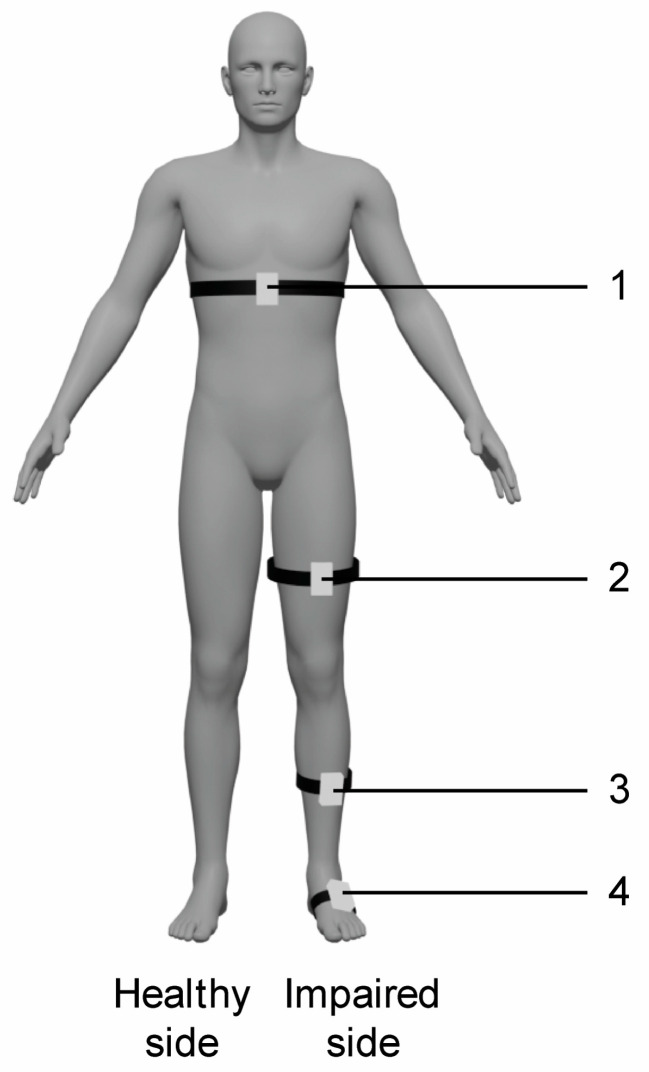
Schematic representation of sensors placement for VR TR trunk and lower limb exercises. Legend: 1. waist (trunk exercises including flexion–extension, trunk rotation, trunk inclination); 2. thigh (hip exercises including flexion–extension, abduction–adduction and stand-to-sit exercise); 2. and 3. thigh and tibia (knee flexion–extension exercise); 3. and 4. tibia and tarsus (dorsiflexion of ankle joint exercise); 4. tarsus (raise on toes exercise).

The experimental training was carried out with the VRRS-HomeKit. The device consists of a tablet placed in a carrying case, equipped with sensors, including K-sensors and K-wand. This system presents a complete set of non-immersive VR exercises to train upper and lower limbs, trunk, and balance. In particular, the patient can be in a seated or in upright position and he/she interacts with 2D scenarios and objects through the wearable sensors. In particular, the K-wand is equipped with light recognition technology used for movement tracking and orientation, which is handled by the patient during catching and reaching virtual exercises for the upper limbs, while the two inertial K-sensors, which are placed on wearable strips of different sizes, are used to carry out full-body motor TR activities. In our rehabilitation protocol, we used the K-sensors to train lower limbs and trunk functions, and they were typically placed on the waist, the upper part of the thigh, and at the tibial level (see Figure 1).

**Figure 2:**
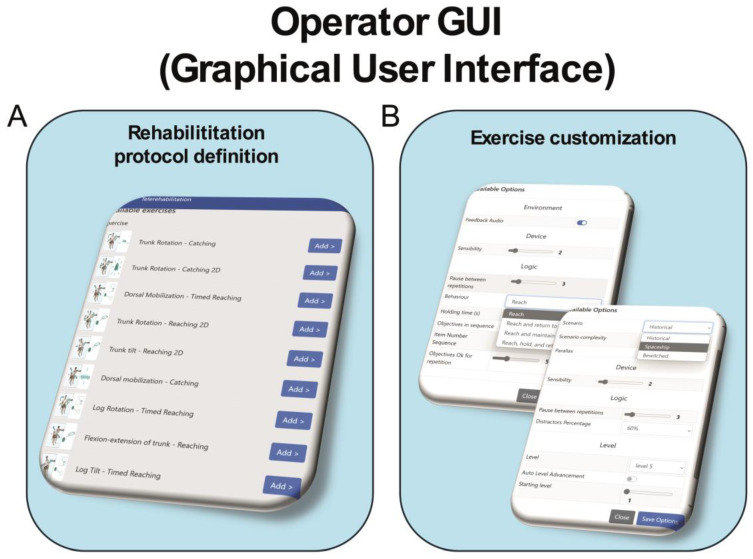
Schematic representation of operator graphical user interface of VRRS-HomeKit system for TR. Legend: Panel (A) displays the graphical interface for defining the rehabilitation protocol, allowing the physiotherapist to easily select the number of exercises. Panel (B) shows the customization board, where the physiotherapist can adjust the settings of the selected exercises, such as the VR scenario, exercise duration, number of repetitions, percentage of distractors (from 0% to 100%), and difficulty level.

Each exercise presents ten levels of difficulty and a distractor percentage ranging from 0 to 100%. During the intervention, the difficulty of the task was adjusted either by the therapist from the graphical user interface (GUI) (see Figure 2) or automatically by the system. Additionally, different scenarios can be selected for each exercise to further enhance the patient’s attention and engagement with the treatment (see Figure 2).

The Tele-Cockpit allows the therapist to take control of the remote device, prescribe treatment, modify exercise parameters and simultaneously see what the patient is doing on the VRRS-HomeKit, fully interacting with them in real time.

Moreover, the TR training sessions lasted about one hour as per the CG, although some extra time (less than 15 min) was also necessary for technology issues, including low bandwidth, content glitches, comfort with the system linked to the distance of the user, or the height at which the device is placed in the home setting and to wear the sensors along the trunk and the lower limb. For these reasons, the presence of a caregiver was essential, as they acted as a “co-therapist” during the TR sessions, assisting patients in completing the sessions.

### 2.7. Statistical Analysis

The data were analyzed using Jamovi software, version 2.4.14 (Jamovi Project, Sydney, Australia), with statistical significance set at *p* < 0.05. Descriptive statistics were presented as median ± interquartile range (IQR), and categorical variables were reported as frequencies and percentages. Given the small sample size and non-normality of distribution, non-parametric tests were employed. Differences between the two groups (EG and CG) in baseline characteristics and clinical assessment scale scores (10MWT, TUG R, TUG L, and MoCA) were assessed using the Mann–Whitney U test. Comparisons of clinical test results between two time points (T0 and T1) were conducted using the Wilcoxon signed-rank test. Due to multiple comparisons in the Wilcoxon signed-rank and Mann–Whitney U tests, the Bonferroni correction was applied, adjusting the significance threshold to α = 0.0125.

## 3. Results

**Figure 3:**
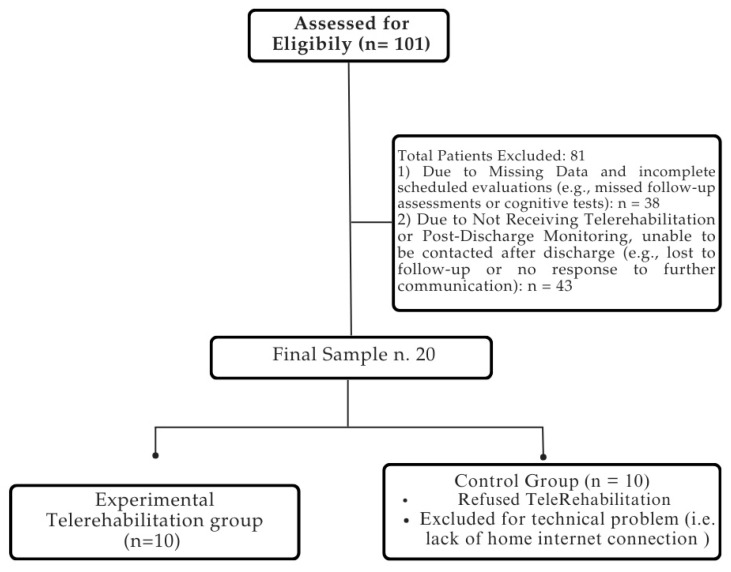
Flowchart of patient selection process. For detailed demographic and clinical characteristics of the sample, please refer to Table 2.

The medical records of 101 patients suffering from stroke were included in the analysis utilizing electronic recovery system data. The final sample consisted of 20 patients, who completed the rehabilitation process without reporting any side effects. The patient selection process is summarized in Figure 3.

No significant differences were found at baseline regarding age (*p* = 0.57), education (*p* = 0.70), and gender (*p* = 1.00). Therefore, the two groups were comparable at the start of the study.

At the end of the training (Table 2), we observed significant improvements in the EG in the 10MWT (*p* = 0.01), TUG L (*p* = 0.01), and MoCA (*p* = 0.005), all of which met the Bonferroni-corrected significance threshold (Table 3).

In the CG, significant improvements were noted for MoCA (*p* = 0.008); however, TUG R (*p* = 0.04) and TUG L (*p* = 0.03) did not meet the corrected significance level (*p* < 0.0125), and the 10MWT did not show significant changes (*p* = 0.16) at the end of training (T1).

These results suggest that, after adjusting for multiple comparisons, the training in the EG led to significant improvements in mobility and cognitive function, except for TUG R (*p* = 0.02), which did not meet the corrected significance threshold (*p* < 0.0125). In contrast, only the MoCA score showed a significant improvement in the CG.

In the between-group analysis (EG vs. CG), no statistically significant differences were observed in any clinical assessment scale at baseline (T0–T0), since they did not achieve the corrected significance threshold (*p* < 0.0125). However, at post-treatment (T1–T1), only the MoCA score showed a statistically significant difference between the two groups (*p* = 0.006) (see Table 4).

## 4. Discussion

The main purpose of our study was to investigate the potential effects of sensor-based virtual TR on lower limb functions in chronic post-stroke patients during the COVID-19 pandemic. Our findings suggest that the EG showed significant improvements in motor function (10MWT, TUG L). Although the CG did not exhibit statistically significant changes, descriptive indicators (e.g., the median) between T0 and T1 suggest a potential trend toward improvement. Furthermore, both groups demonstrated significant improvements in global cognitive function (MoCA).

In line with our previous study on severe acquired brain injury, we found that balance and gait functions improved after the TR treatment with a non-immersive VR device, as well as cognitive functions [19].

The current literature is mostly focused on TR for upper limb and cognitive training [24,25,26], but only a few studies investigated the effects of lower limb functions, including gait and balance [6,7,27,28]. Lower limb functions, such as gait and balance, are often impaired in post-stroke patients. Stroke survivors usually have a decreased stance phase and increased swing phase on the paretic side, during gait [29]. In addition, the walking speed is also reduced, and the stride length is shorter [29]. Altogether, these abnormalities within the muscle weakness increase the risk of falls in this patient population [30]. Thus, improving walking safety and speed is one of the major goals for stroke rehabilitation to prevent falls and indirectly improve the quality of life. According to Su et al. [31], post-stroke patients could benefit from TR for balance training, including stand-to-sit exercises, as those proposed in our protocol. However, some authors are still questioning the efficacy of TR on gait outcomes. For example, Deng et al. [27] used the 10MWT and did not find statistically significant improvements, though they reported positive changes in ankle dorsiflexion. In contrast, Chen et al. [7] and Wu et al. [32] reported significant improvements in TUG performance in TR groups compared to controls. According to a recent systematic review [31], which analyzed eight studies on telerehabilitation for post-stroke gait recovery, TR was found to be an effective delivery method to improve balance and functional mobility, with results comparable or even superior to conventional home-based rehabilitation. These findings are consistent with our results, especially regarding improvements in gait performance.

One possible explanation for our results could be placed in the delivery of care devices. Deshmukh et al. [28], found a great heterogeneity among the TR devices, ranging from simple videoconferencing platforms to smartphones, whereas only Lloréns et al. [33] used a TR VR-based device (Kinect) reporting promising results for the reacquisition of balance and locomotor skills.

VR provides augmented feedback (i.e., audio-visual stimuli) that enhances the participation and the motivation, which are fundamental to the success of the rehabilitation. VR systems constantly inform patients of their progress and rehabilitation status through immediate auditory, visual, and textual feedback [30,31]. This real-time feedback reinforces learning by providing clear information on exercise outcomes. Additionally, the VRRS Homekit includes an integrated reward-based scoring mechanism (e.g., performance-based points), which has been shown to enhance motivation and promote emotional engagement, such as increased self-esteem and satisfaction [34,35,36]. These elements may help explain the cognitive improvements observed in our study. However, this internal scoring system is an inherent feature of the training environment and was not employed as an outcome measure in the present study. In the VR environment, exercises promote awareness of movement outcomes and performance quality [14]. In this way, VR can stimulate the central nervous system, which receives increased feedback signals (augmented feedback), inducing profound changes in neural plasticity, responsible for motor and cognitive function recovery [14].

Interestingly, both the EG and CG showed global cognitive improvements measured by MoCA. These gains may reflect the close relationship between motor and cognitive domains, especially the connection between executive functions and balance [37]. Notably, our findings suggest that the integration of sensor-based VRRS training with a dual-task component, such as performing motor tasks while simultaneously avoiding distractors, may enhance cognitive performance in chronic post-stroke patients. Studies have shown that lower limb muscle strength is correlated with executive function, suggesting that physical training can support cognitive performance [33,34]. For instance, Frith et al. [38] found that older adults who trained lower limb strength had better executive functioning and a 34% reduced risk of cognitive decline. Further studies are needed to clarify the specific contribution of lower limb strength and balance to cognitive outcomes. These findings are consistent with broader evidence linking executive function closely to balance, and showing moderate to mild associations between motor abilities and both processing speed and episodic memory [38,39,40,41,42,43]. Nevertheless, the benefits of physical activity on cognition [44] should not be overlooked, as increased cerebral blood flow, improved quality of life, and preservation of gait speed are all safeguards against mental deterioration. Another factor that may have contributed to the cognitive improvements observed in the EG is the use of VR. VR-based rehabilitation is thought to stimulate central nervous system through augmented feedback mechanisms, thereby promoting neuroplastic changes essential for cognitive recovery [45,46].

Our findings suggest the adoption of VR-based TR as a key component of the stroke recovery path. By simultaneously addressing both motor and cognitive deficits, this approach may enhance functional outcomes and contribute to a better quality of life. Future research should more objectively explore the roles of lower limb strength and balance in cognitive recovery. The neurocognitive benefits of physical activity are widely supported by the literature, with mechanisms including enhanced vascular function, improved mobility, and overall psychological well-being, factors that contribute to long-term protection against cognitive decline [47,48,49].

Another important aspect is the presence of the caregiver during TR sessions. In our previous randomized controlled trial involving patients with severe acquired brain injury [19], we observed that home-based TR with non-immersive VR led to significant improvements in both motor and cognitive functions in patients, and concurrently reduced the burden experienced by caregivers. In that context, the caregiver acted as a co-therapist and received structured support from a multidisciplinary team. In contrast, the present study focused on chronic stroke patients and did not assess caregiver-related outcomes, as this was beyond the scope of our objectives. Nevertheless, caregivers were actively involved during the intervention, likely contributing to treatment feasibility and adherence, particularly by assisting with the home setup and helping patients overcome barriers to accessing care. Future studies should examine the specific contribution of caregiver involvement not only to treatment feasibility and emotional outcomes but also to the clinical effectiveness of home-based VR telerehabilitation in stroke populations.

Finally, it is essential to highlight the role of TR in addressing the challenges imposed by the COVID-19 pandemic. Restrictions on in-person interactions and the need for social distancing severely limited access to conventional stroke rehabilitation services, exposing the vulnerability of traditional care models [8,9]. Given the critical importance of motor rehabilitation in preventing long-term physical and cognitive decline after stroke, remote interventions, such as TR, became a necessary alternative. They enabled the monitoring and treatment of chronic or recently discharged patients who might otherwise have been left without adequate follow-up [50]. Beyond its relevance in emergency contexts, TR has demonstrated its potential as a safe and sustainable solution to ensure the continuity of care, reducing barriers, enhancing access, and promoting functional recovery without direct clinician–patient contact [9].

### Limitations and Strengths of the Study

Our study has some limitations that need to be acknowledged. Its retrospective design limits the ability to control for all confounding variables. The small sample size reduces statistical power and increases the risk of type II error, potentially leading to an underestimation of the true effects of the intervention. These factors also limit the generalizability of the findings to the broader post-stroke population.

Moreover, the lack of objective gait analysis and the absence of kinematic and kinetic data from inertial sensors may have constrained the precision of motor outcome evaluation. All participants were on pharmacological treatment, which could have influenced both motor and cognitive results; therefore, our findings may not be generalizable to stroke patients with different therapeutic profiles.

Additionally, minor baseline differences in clinical scores (e.g., MoCA, 10MWT, TUG) may reflect interindividual variability not fully controllable in a retrospective framework. While these differences did not reach statistical significance, we acknowledge that they could have influenced the post-treatment outcomes. Consequently, we cannot exclude the possibility that part of the observed improvements, particularly in the EG, may be attributable, at least in part, to pre-existing clinical differences between the groups. Important clinical variables, such as stroke severity, time since onset, or use of assistive devices, were not systematically recorded and could not be included in the analysis. These limitations, inherent to retrospective data, should be taken into account when interpreting our results.

Despite these constraints, the present study provides valuable exploratory insights, emphasizing the need for future prospective trials with larger, more homogeneous and stratified samples, as well as refined outcome measures. Such studies will be essential to determine whether the observed effects are partially or entirely attributable to the innovative TR approach.

Unlike our previous randomized controlled trial on TR in patients with severe acquired brain injury [19], which evaluated both patient progress and caregiver burden, the current study introduces several novel aspects. It focuses specifically on lower limb (e.g., both gait and postural stability) and cognitive outcomes in chronic stroke patients and implements a dual-task protocol using a sensor-based VR system designed for home use. Although caregiver-related effects were not formally assessed, the active involvement of caregivers and the home-based nature of the intervention suggest potential indirect benefits that warrant future investigation. Therefore, the originality of this study lies in its application of an ecologically valid, technology-driven rehabilitation model that integrates motor and cognitive training through a dual-task VR approach, tailored to real-world, post-pandemic clinical conditions.

## 5. Conclusions

In our study, we highlighted the role of TR in chronic stroke patients for improving lower limb function, suggesting that this approach is not inferior to conventional home-based rehabilitation. TR can be a valuable approach in rehabilitation, as it enables innovative and comprehensive treatment to be delivered directly to the patient’s home. In this situation, the patient receives guidance both from the therapist and from their caregiver and can be easily trained at home, overcoming geographic and economic barriers. Larger prospective studies are needed to confirm our promising findings and to investigate if and to what extent motor outcomes last.

## Figures and Tables

**Table 1:** Detailed description of the performed exercises for both groups (EG and CG) and of sensor placement for patients in the EG.

Exercise’s Goal	EG—Sensor-Based Non-Immersive Virtual Reality Telerehabilitation	CG—Conventional Home-Based Rehabilitation
Stand-to-sit (bilateral exercise)	Catching: The patient gets up and sits down from a chair by catching objects on the screen (such as fruit, raindrops, clothes, airplanes), taking care to avoid distractors. Reaching: The patient gets up and sits down from the chair by reaching objects on the screen as quickly as possible.	Sit-to-stand exercise and squats were used in order to return to the sitting position with the manual assistance provided by a therapist.
Raise on toes (bilateral exercise)	Holding onto a solid base, the patient stands up on their toes and lowers themselves by picking up and reaching for objects on the screen.	The patient, holding on to a solid base, stands up on his toes and then returns to the starting position, under the supervision and guidance of the therapist.
Flexion–extension of hip joint	Catching: the patient is seated in front of the screen, and he/she catches the objects in different VR scenarios (i.e., underwater, forest, sky, bedroom) with the flexion or the extension of the hip, avoiding distractors. Reaching: the patient is seated in front of the screen, and he/she reaches the objects (e.g., balls) with the flexion or the extension of the hip.	The patient in a sitting position is stimulated by the therapist to actively perform hip flexion–extension trying to reach the targets (e.g., ball, colored points) indicated by the therapist.
Flexion–extension of knee joint	Catching: the patient is seated in front of the screen, and he/she catches the objects in different VR scenarios (i.e., underwater, forest, sky, bedroom) with the flexion or the extension of the knee, avoiding distractors. Reaching: the patient is seated in front of the screen, and he/she reaches the objects (e.g., balls) with the flexion or the extension of the knee.	The patient in a sitting position is stimulated by the therapist to actively perform knee flexion–extension trying to reach the targets (e.g., ball, colored points) indicated by the therapist.
Dorsi-flexion of ankle joint	Catching: the patient is seated in front of the screen, and he/she catches the objects in different VR scenarios (i.e., underwater, forest, sky, bedroom) with the dorsi-flexion of the ankle, avoiding distractors. Reaching: the patient is seated in front of the screen, and he/she reaches the objects (e.g., balls) with the dorsi-flexion of the ankle.	The patient is seated and dorsiflexes the ankle to reach the targets (e.g., ball, colored points) identified by the therapist.
Abduction–adduction of hip joint	Catching: the patient is seated in front of the screen, and he/she catches the objects in different VR scenarios (i.e., underwater, forest, sky, bedroom) with the abduction or the adduction of the hip, avoiding distractors. Reaching: the patient is seated in front of the screen, and he/she reaches the objects (e.g., balls) with the abduction or the adduction of the hip.	The patient in a sitting position is stimulated by the therapist to actively perform hip flexion–extension trying to reach the target (e.g., ball, colored points) indicated by the therapist.
Flexion–extension of trunk	Catching: the patient is seated in front of the screen, and he/she catches the objects in different VR scenarios (i.e., underwater, forest, sky, bedroom) moving their trunk forward (flexion) or backwards (extension), avoiding distractors. Reaching: the patient is seated in front of the screen, and he/she reaches the objects (e.g., balls), moving their trunk forward (flexion) or backward (extension).	The patient is seated in front of the therapist, and he/she holds a stick or ball, moving the trunk forward and backward.
Trunk rotation	Catching: the patient is seated in front of the screen, and he/she catches the objects in different VR scenarios (i.e., underwater, forest, sky, bedroom) rotating the trunk from one side to the other side and avoiding distractors. Reaching: the patient is seated in front of the screen, and he/she reaches the objects (e.g., balls) rotating the trunk from one side to the other side and avoiding distractors.	The patient is seated in front of the therapist, and he/she holds a stick or ball, rotating the trunk from side to side.
Trunk inclination	Catching: the patient is seated in front of the screen, and he/she catches the objects in different VR scenarios (i.e., underwater, forest, sky, bedroom) bending the trunk on one side and on the other side and avoiding distractors. Reaching: the patient is seated in front of the screen, and he/she reaches the objects (e.g., balls), bending the trunk on one side and on the other side.	The patient is seated in front of the therapist, and he/she holds a stick or ball, bending the trunk from side to side.

**Table 2:** Demographic characteristics at baseline for both groups.

	Experimental	Control	All	*p*-Value
Participants	10	10	20	
Age	54.3 ± 10.2	58.1 ± 13.5	56.2 ± 11.8	0.57
Education	12.8 ± 3.0	12.0 ± 3.9	12.4 ± 3.4	0.70
Gender: - Male - Female	6 (60%) 4 (40%)	6 (60%) 4 (40%)	12 (60%) 8 (40%)	1.00
Aetiology: - Haemorrhagic - Ischemic Affected side: - Left - Right	5 (50%) 5 (50%) 6 (60%) 4 (40%)	5 (50%) 5 (50%) 6 (60%) 4 (40%)	10 (50%) 10 (50%) 12 (60%) 8 (40%)	1.00 1.00

Legend: Quantitative variables were expressed as means ± standard deviations, categorical variables as frequencies, and percentages. *p*-value U- Mann–Whitney < 0.05.

**Table 3:** Wilcoxon Rank test of clinical scores between baseline (T0) and follow-up (T1), for both experimental (EG) and control (CG) groups.

Clinical Assessment	Experimental Group		*p*-Value	Control Group		*p*-Value
	T0	T1		T0	T1	
10MWT	6.51 (5.70–8.04)	5.79 (5.14–7.60)	**0.010**	13.7 (6.8–17.9)	10.7 (8.2–15.0)	0.16
TUG R	10.2 (8.40–18.0)	10.0 (7.94–16.2)	0.02	26.5 (21.3–34.0)	24.5 (21.1–32.9)	0.04
TUG L	10.7 (8.59–18.0)	10.1 (7.81–17.3)	**0.010**	25.3 (18.4–35.6)	24.2 (18.1–33.8)	0.03
MoCA	24.0 (23.0–24.8)	25.5 (25.0–27.0)	**0.005**	21.5 (21.0–22.0)	23.0 (22.0–23.0)	**0.008**

Legend: Scores are in median (first-third quartile); significant differences are in bold. Bonferroni correction < 0.0125. Legend: 10MWT (10-m walking test); TUG R (Timed-Up-Go right); TUG L (Timed-Up-Go left); MoCA (Montreal Cognitive Assessment).

**Table 4:** Statistical comparison (*p*-values) of clinical assessment scale scores from baseline (T0) to post-treatment (T1) between the experimental and control groups.

Clinical Assessment Scales	Experimental Group vs. Control Group T0—T0	Experimental Group vs. Control Group T1—T1
10MWT	0.14	0.08
TUG R	0.03	0.03
TUG L	0.07	0.08
MoCA	0.05	**0.006**

Legend: *p*-values are calculated using the Mann–Whitney U test; significant differences are in bold. Bonferroni correction <0.0125. Legend: 10MWT (10-m walking test); TUG R (Timed-Up-Go right); TUG L (Timed-Up-Go left); MoCA (Montreal Cognitive Assessment).

## Data Availability

Data will be available on request to the corresponding author due to privacy reasons.

## Abbreviations

CG	control group
COVID-19	Coronavirus Disease 2019
EG	experimental group
FFP3	Filtering Face Piece class 3
GUI	graphical user interface
IQR	interquartile range
MAS	Modified Ashworth Scale
MoCA	Montreal Cognitive Assessment
T0/T1	Timepoint 0/Timepoint 1 (pre- and post-treatment)
TR	telerehabilitation
TUG L	Timed-Up-Go Left
TUG R	Timed-Up-Go Right
VR	virtual reality
VRRS	Virtual Reality Rehabilitation System
10MWT	10-m walking test
